# Flaviviruses in Europe: Complex Circulation Patterns and Their Consequences for the Diagnosis and Control of West Nile Disease 

**DOI:** 10.3390/ijerph10116049

**Published:** 2013-11-12

**Authors:** Cécile Beck, Miguel Angel Jimenez-Clavero, Agnès Leblond, Benoît Durand, Norbert Nowotny, Isabelle Leparc-Goffart, Stéphan Zientara, Elsa Jourdain, Sylvie Lecollinet

**Affiliations:** 1 UMR1161 Virologie INRA, ANSES, ENVA, EU-RL on equine West Nile disease, Animal Health Laboratory, ANSES, Maisons-Alfort 94704, France; E-Mails: cecile.beck@anses.fr (C.B.); stephan.zientara@anses.fr (S.Z.); 2 CISA-INIA, Valdeolmos (Madrid) 28130, Spain; E-Mail: majimenez@inia.es; 3 Département Hippique, VetAgroSup, Marcy l’Etoile 69280, France; E-Mail: agnes.leblond@vetagro-sup.fr; 4 UR346, INRA, Saint Genès Champanelle 63122, France; E-Mail: elsa.jourdain@clermont.inra.fr; 5 Epidemiology Unit, Animal Health Laboratory, ANSES, Maisons-Alfort 94704, France; E-Mail: benoit.durand@anses.fr; 6 Viral Zoonoses, Emerging and Vector-Borne Infections Group, Institute of Virology, University of Veterinary Medicine Vienna, Vienna 1210, Austria; E-Mail: norbert.nowotny@vetmeduni.ac.at; 7 Department of Microbiology and Immunology, College of Medicine and Health Sciences, Sultan Qaboos University, Muscat 123, Sultanate of Oman; 8 IRBA, Marseille 13384, France; E-Mail: isabelle.leparcgoffart@gmail.com

**Keywords:** flaviviruses, West Nile virus, antibodies, cross-reactivity, diagnosis, cross-protection, ADE, natural infection, vaccination, vector-borne diseases

## Abstract

In Europe, many flaviviruses are endemic (West Nile, Usutu, tick-borne encephalitis viruses) or occasionally imported (dengue, yellow fever viruses). Due to the temporal and geographical co-circulation of flaviviruses in Europe, flavivirus differentiation by diagnostic tests is crucial in the adaptation of surveillance and control efforts. Serological diagnosis of flavivirus infections is complicated by the antigenic similarities among the *Flavivirus* genus. Indeed, most flavivirus antibodies are directed against the highly immunogenic envelope protein, which contains both flavivirus cross-reactive and virus-specific epitopes. Serological assay results should thus be interpreted with care and confirmed by comparative neutralization tests using a panel of viruses known to circulate in Europe. However, antibody cross-reactivity could be advantageous in efforts to control emerging flaviviruses because it ensures partial cross-protection. In contrast, it might also facilitate subsequent diseases, through a phenomenon called antibody-dependent enhancement mainly described for dengue virus infections. Here, we review the serological methods commonly used in WNV diagnosis and surveillance in Europe. By examining past and current epidemiological situations in different European countries, we present the challenges involved in interpreting flavivirus serological tests and setting up appropriate surveillance programs; we also address the consequences of flavivirus circulation and vaccination for host immunity.

## 1. Introduction

West Nile virus (WNV) is a widespread re-emerging pathogen that belongs to the *Flaviviridae* family and *Flavivirus* genus and is one of the most threatening flaviviruses in Europe (for a recent review see [1]). This arbovirus is transmitted by mosquitoes in a cycle in which different species of birds act as reservoir hosts, amplifying the virus. Spillover from this cycle occasionally occurs and may cause West Nile disease in mammalian hosts. Horses and humans may be particularly affected, which is a matter of great concern to the veterinary and public health authorities of countries with West Nile cases. Although mammals are susceptible to WNV infection, most species are regarded as dead-end hosts; WNV does not efficiently replicate within their cells and they cannot transmit WNV to new vectors [2]. 

Most WNV infections are asymptomatic in horses and humans or are associated with an influenza-like illness (characterized by moderate to high fever, weakness, and myalgia). Only infrequently, in less than 1% infections in humans and 10% of infections in horses, do acute meningitis, encephalitis, or flaccid paralysis develop (the latter has only been reported in humans); neurological symptoms and lesions are not specific to WNV infections [3]. Consequently, laboratory tests are essential to confirm or exclude WNV infection. Because of the virus’ low-level and short-term viremia in humans and horses as well as the late appearance of clinical signs when the viremic phase is over, the primary tools used to diagnose WNV consist of indirect or serological tests that aim to detect WNV antibodies. Rapid and high-throughput assays that do not require the use of infectious virus, such as ELISAs, hemagglutination-inhibition tests (HITs) or immunofluorescence assays (IFAs), are usually preferred (see Section 2.2). However, seropositivity has to be interpreted with care because of the frequent cross-reactions among flaviviruses observed in these tests; results should be systematically confirmed by comparative virus neutralization tests (VNTs) that use a panel of viruses known to circulate in the area under investigation [4,5]. Accordingly, serological tools have to be adapted to specific epidemiological situations involving WNV. 

Since WNV was introduced into New York City in 1999, it has rapidly diffused throughout North America. It has infected tens of thousands of humans (>36,800) and horses (>25,000) according to the Centers for Disease Control and Prevention [6] and resulted in widespread bird mortality, causing dramatic declines in some wild bird species (e.g., American crows, *Corvus brachyrhynchos*) [7,8,9]. In stark contrast, in Europe, where the disease is re-emerging, WNV has caused only sporadic clinical cases and self-limited outbreaks until 2007, with no or isolated incidents of wild bird mortality [10,11]. Several non-mutually exclusive hypotheses have been proposed to explain the different patterns observed on the different continents. They each address one of the four primary components of the WNV epidemiological cycle: The virus, the host, the vector, and the environment. First, it has been suggested that the intrinsic virulence of WNV strains circulating in America and Europe could differ, with the North American strain being more virulent for birds [11,12,13]. Second, Palearctic versus Nearctic wild bird species have been hypothesized to differ in their susceptibility to WNV infection, with the former having co-evolved with WNV while the latter were initially naive hosts [14]. Third, mosquitoes found in North America and in Europe may differ in their competence as vectors [15]. Finally, the last hypothesis stresses the importance of past exposure to various flaviviruses; exposure to the wide diversity of flaviviruses circulating in Europe, and more generally in the western Palearctic [11,16], might confer partial or complete cross-protection to WNV (see Section 3 for further discussion). Other ecological factors could also, to some extent, account for these different WNV epidemiological patterns. In particular, biodiversity or species richness could potentially act as protective factors via a “dilution effect” or, conversely, they may facilitate WNV transmission [17,18,19].

The differences in West Nile virus transmission and clinical impact in Europe versus North America imply that diverse and adapted approaches for identifying and controlling WNV infections should be employed. Even within the Euro-Mediterranean, WNV outbreak frequency and severity decrease from east to west (eCDC [20,21]). As a consequence, intervention measures based on experience gained in distant or even neighbouring transmission areas can be misleading, and a detailed description of the eco-epidemiology of a particular WNV outbreak is needed to provide tailored and balanced public health responses. For instance, surveillance of avian mortality proved very useful in North America (where mass wild bird mortality events usually preceded human and domestic animal cases), but it has not yet been useful in Europe, with the exception of some of the European regions experiencing WNV lineage 2 circulation [22,23,24]. Passive or clinical surveillance of humans and horses as well as serological surveillance of wild birds or equines have been far more instructive in Europe, and their use should be considered by health authorities [25,26]. Moreover, entomological surveillance effectively predicts WNV activity and outbreaks, even though mosquitoes in Europe have been found to have lower infection rates than mosquitoes in the U.S. [27,28].

Similar reasoning should be applied with respect to WNV diagnostic tools: Their validity in each specific WNV epidemiological situation should be verified to ensure that infections caused by varied WNV strains are detected [29]. This process is particularly important in Europe or Africa, where several genetic lineages circulate in overlapping areas; in contrast, only a single-lineage WNV strain (1a) was introduced into North America [30,31,32,33,34,35]. Furthermore, diagnostic tools should be specific enough to avoid detecting cross-reactions with closely related flaviviruses that may be circulating in the same areas. Many flaviviruses belonging to the Japanese Encephalitis serocomplex have a wide distribution range, including West Nile virus (WNV), Saint Louis encephalitis virus (SLEV) in the Americas, Murray Valley encephalitis virus (MVEV) in Australia, Japanese encephalitis virus (JEV) in southern Asia and Oceania, and Usutu virus (USUV) in Europe and Africa [36]. More specifically, in central and eastern Europe, the high prevalence of tick-borne encephalitis virus (TBEV) and human TBEV vaccination campaigns [37] frequently result in serological cross-reactions when routine diagnostic tests such as WNV ELISAs or IFAs are conducted [38,39,40,41]. The interpretation of serological test results may be even more problematic when investigating infections caused by flaviviruses belonging to the same serocomplex, such as WNV and USUV in Europe, since cross-neutralization will also be observed. In such situations, comparative VNTs that include every flavivirus suspected to circulate within a given area are called for. Therefore, prior knowledge of flavivirus diversity at the local level is essential before rigorous serological surveys can be undertaken. In the first section of this review, we assess current flavivirus diversity and the risk of future flaviviruses being introduced into Europe, which could impact the accuracy of WNV serological diagnoses. In the second section, we outline the challenges involved in interpreting the diverse range of WNV serological tests and setting up appropriate surveillance programs in reservoir or susceptible hosts. In the final section, we address the consequences of past exposure to flaviviruses for WNV immunity. Most studies investigating flavivirus cross-protection or infection facilitation have found that prior infection or vaccination with heterologous flaviviruses should provide partial protection against WNV, with more efficient protection being provided by more closely related viruses. 

## 2. Flavivirus Diversity in Europe

The *Flavivirus* genus comprises 53 viruses (ICTV [42]). Many of them are human pathogens of concern, such as the viruses that cause dengue (DENV), yellow fever (YFV), Japanese encephalitis (JEV), West Nile (WNV), or tick-borne encephalitis (TBEV); they are transmitted by mosquitoes (DENV, YFV, JEV, WNV) or ticks (TBEV) [43,44]. 

**Figure 1:**
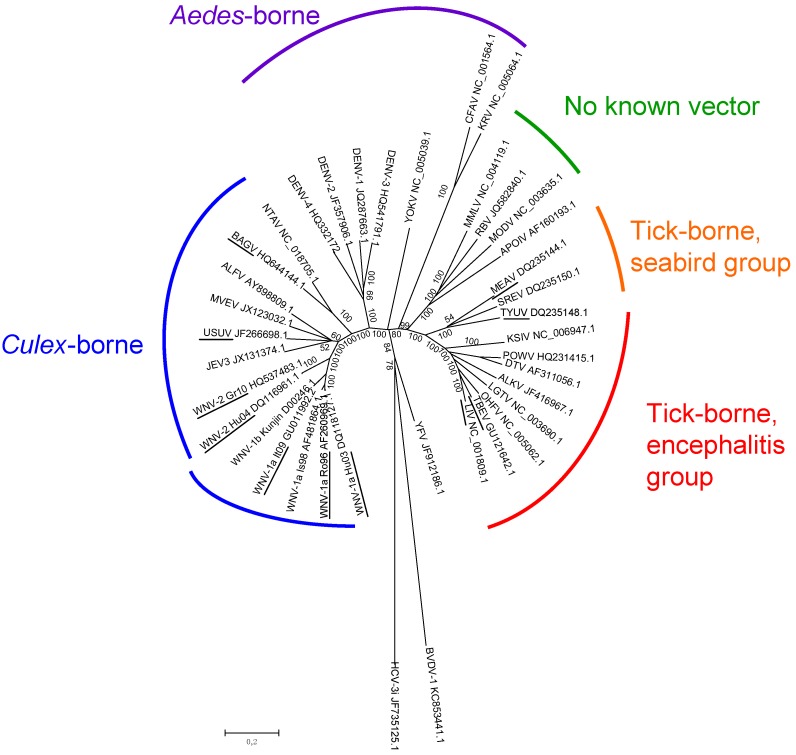
Genetic relatedness of flaviviruses, evaluated using genetic alignments of complete genomic sequences. GenBank accession numbers are indicated on the tree branches of each virus.

Early attempts to define flavivirus relatedness were based on antigenic cross-reactivity in VNTs, HITs, and complement fixation tests (CFTs). Albeit imprecise, serological studies allowed different serocomplexes to be defined, including the JEV (WNV and USUV in Europe), YFV, DENV, and Ntaya virus (Bagaza virus—BAGV—in Europe) serocomplexes [5,45]. Molecular characterization of the flavivirus RNA genome allowed the precise taxonomic classification of flaviviruses and the study of their genetic evolution and dispersal [44,46,47]. Three distinct groups of flaviviruses were identified: tick-borne viruses, mosquito-borne viruses, and viruses with unknown vectors [47]. Mosquito-borne viruses can be further subdivided into *Culex* and *Aedes* clades, which also differ in their vertebrate hosts and pathogenesis (Figure 1). *Culex*-clade viruses have bird reservoirs, are neurotropic, and frequently cause meningo-encephalitis, while *Aedes*-clade viruses have primate reservoirs, are non-neurotropic, and mainly result in hemorrhagic diseases [43,48]. The tick-borne viruses also form two groups: One group circulates among seabirds, while the other, the tick-borne encephalitis group, is primarily associated with rodents. This latter group generally produces encephalitic disease, although Omsk Hemorrhagic Fever virus (OHFV) and Kyasanur Forest Disease virus (KFDV) also cause hemorrhagic diseases in humans. Interestingly, tick-borne flaviviruses seem to evolve at a slower rate than mosquito-borne flaviviruses, probably as a result of a slower virus replication rate in ticks and longer generation times of their tick hosts [49]. 

WNV: West Nile virus, JEV: Japanese encephalitis virus, USUV: Usutu virus, MVEV: Murray Valley encephalitis virus, ALFV: Alfuy virus, NTAV:Ntaya virus, DENV: Dengue virus, YOKV: Yokose virus, CFAV: Cell Fusing Agent virus, KRV: Kamiti River virus, MMLV: Montana Myotis Leukoencephalitis virus, RBV: Rio Bravo virus, MODV: Modoc virus, APOIV: Apoi virus, MEAV: Meaban virus, SREV: Saumarez Reef virus, TYUV: Tyuleniy virus, KSIV: Karshi virus, POWV: Powassan virus, DTV: Deer Tick virus, ALKV: Alkhurma virus, LGTV: Langat virus, OHFV: Omsk Hemorrhagic Fever virus, TBEV: tick-borne encephalitis virus, LIV: Louping Ill virus, YFV: Yellow Fever virus. 2 outgroups were used, HCV for Hepatitis C virus (*Hepacivirus* genus, *Flaviviridae* family) and BVDV for Bovine Viral Diarrhea virus (*Pestivirus* genus, *Flaviviridae* family). Flaviviruses and WNV strains isolated in Europe are underlined. The phylogenetic tree was constructed using neighbor-joining with Jukes–Cantor parameter distances (scale bar) in MEGA, version 5.2 [50]. A bootstrapped confidence interval (1000 replicates) and a confidence probability value based on the standard error test were also calculated using MEGA.

**Figure 2:**
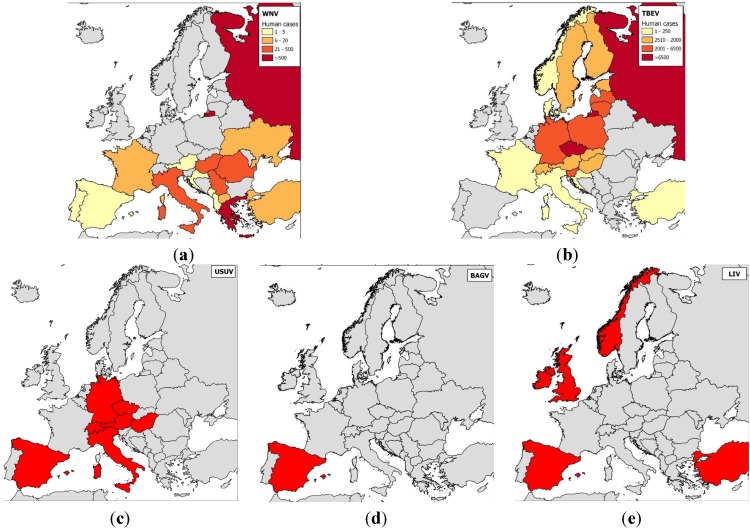
Maps of the distributions of the main flaviviruses found in Europe.

Notes: (**a**) WNV—human cases were aggregated across a 10-year period (2003–2012, data obtained from the European Centre for Disease Prevention and Control (eCDC), World Organization for Animal Health (OIE), and scientific publications) and the numbers of cases per country are indicated; (**b**) TBEV—human cases were aggregated across a 10-year period (2000–2009) and the numbers of cases per country are indicated [51,52]; (**c**) USUV; (**d**) BAGV; and (**e**) LIV. For USUV, BAGV, and LIV, countries that reported clinical cases in wild birds (USUV, BAGV) or sheep, goats or horses (LIV) are highlighted in red.

Many antigenically-related flaviviruses have been identified, and mosquito-borne flaviviruses have been found on every continent except Antarctica. Similarities in disease symptoms—only two syndromes, encephalitic and hemorrhagic, have been described in humans and animals—make it impossible to differentiate flavivirus infections clinically and precisely identify circulating flaviviruses. In the next subsection, we will describe flavivirus circulation and introduction risk in Europe (Figure 2).

### 2.1. Epidemiology of *Flavivirus* Infections in Europe

Tick-borne flaviviruses are among the most medically important arboviruses in Europe. TBEV severely infects more than 10,000 humans every year and is transmitted by *Ixodes ricinus* and *I. persulcatus*; most cases have occurred in central Europe (Western subtype), Finland, and the European part of Russia (Western and Siberian subtypes) [53] (Figure 2). Tick-borne flaviviruses also comprise the louping-ill virus (LIV) and OHFV. Louping-ill is a long-known disease of sheep [54] that has clear zoonotic potential. LIV is found in Norway, Spain, Ireland, Turkey, and Bulgaria, although it most commonly occurs on British Islands [53]; it is transmitted by *I. ricinus* ticks. OHFV, found in Russia, cycles between rodents (e.g., narrow-headed voles, *Microtus gregalis* or water voles, *Arvicola terrestris*) and *Dermacentor reticulatus* ticks in western Siberia, causing hemorrhagic diseases in humans and in some rodents (muskrats, *Ondatra zibethicus*) [55]. 

Several other tick-borne flaviviruses have thus far not been found to cause disease in humans or animals, and their potential pathogenicity in humans and animals is consequently unknown. Meaban virus (MEAV), which is transmitted by soft *Ornithodoros maritimus* ticks among seabirds and was first identified in Brittany, France, is one such flavivirus [56]. Tyuleniy virus (TYUV) also belongs to the seabird tick-borne flavivirus group and has been isolated from ticks on the Atlantic coast of France, the Lofoten Islands (Norway), the western U.S. coast, and northern Russia. In contrast to MEAV, TYUV has been implicated in three cases of human illness so far [57]. 

A wider diversity of tick-borne flaviviruses has been detected in Europe than in North America; the Powassan virus is the major tick-borne flavivirus found to be pathogenic in humans in North America. Mosquito-borne flaviviruses are similarly more diverse in Europe than in North America; specifically, WNV and USUV are widely distributed throughout Europe, and BAGV was identified in Spain [58]. 

Of all the mosquito-borne flaviviruses, WNV has the most widespread geographical distribution and the largest vector and host range [58]. It was first described in Europe in the 1960s when seropositive animals or virus isolates were discovered in France, Portugal, and Cyprus [59,60]. WNV has historically been considered less pathogenic in humans than DENV or YFV. However, more virulent genotypes have emerged since 1998: Isolates from Israel (1998), Hungary (2003), or North America (1999) belonging to the Israelo-American cluster of WNV lineage 1a are highly pathogenic in birds and mammals [8,31,61], and lineage 2 viruses have caused an increasing number of WNV outbreaks in Europe since 2008 [62,63]. In Europe, WNV has mainly been reported in central and south-eastern Europe, regions in which WNV infections and virulence have recently increased, and the implicated viruses have spread to new areas, including Bulgaria and Greece in 2010, Albania and Macedonia in 2011, and Croatia, Serbia, and Kosovo in 2012. Accordingly, alarming outbreaks were reported in several European countries in 2010; 261 confirmed human cases, including 34 deaths, occurred in Greece, 57 cases and five deaths occurred in Romania, and 480 cases and six deaths occurred in Russia [34,62,64]. Another European country, Italy, has also experienced numerous WNV epidemics caused by genetically divergent isolates; starting in 2008 and 2010, lineage 1 and 2 strains respectively caused outbreaks at locations across the country (e.g., Emilia-Romania in the northeast, Tuscany, Sardinia, and Lazio in the center, and Sicily and Molise in the south) [27,65]. 

USUV is a flavivirus that originated in Africa and is closely related to WNV. It is transmitted by *Culex* mosquitoes and was most probably introduced into Europe by migrant birds; retrospectively, it was first identified in cases of wild bird die-offs in 1996 in Tuscany, Italy [66]. In contrast to WNV, it rarely causes severe neurological symptoms in humans, but virulent strains have caused considerable bird mortality, especially in blackbirds (*Turdus merula*) in central Europe, starting in Austria in 2001 [67,68]. USUV is known to circulate in Hungary, Switzerland, Italy, Spain, the Czech Republic and Germany [69,70,71,72,73,74,75], and USUV neutralizing antibodies were recently found in animals in France and Croatia [76,77].

BAGV is identical to Israel turkey meningoencephalitis virus (ITMV), which was first described in Israel in 1960 based on samples taken from neurologically afflicted turkeys. It has more recently been found in infected partridges and pheasants in southern Spain [78]. Anti-BAGV antibodies were recently found in the sera of encephalitic patients in India, underscoring the virus’ zoonotic potential [79]. 

Far more flaviviruses have been identified in recent years in Europe. For instance, flaviviruses that exclusively infect insects were discovered in Italy and Spain [80,81], a JEV or a JEV-like virus was found in *Culex pipiens* in northern Italy [82], and Lammi virus, a new flavivirus phylogenetically related to *Aedes*-borne viruses, was identified in Finland [83]. The main concern with the large and expanding diversity of flaviviruses in Europe is that more and more regions are reporting co-circulation of different flaviviruses; for instance, WNV and USUV co-circulate in northeastern Italy [84] and BAGV and WNV co-circulate in southern Spain [85]. Future introductions of tick-borne and mosquito-borne flaviviruses should be anticipated, and the expanding range of flavivirus vectors should be taken into account. Several risk-assessment studies have examined potential introduction routes and scenarios using information on reservoir hosts or infected vectors [86,87,88,89,90]. Among all the flavivirus vectors, *Aedes albopictus*, which vectors DENV and Zika virus, provides a striking example of the successful invasion of new territories [91,92]; it was responsible for autochthonous DENV infections in France and Croatia in 2010 [93,94]. 

### 2.2. Vaccination Programs in Europe

In Europe, a few human vaccines against flaviviruses are available, specifically those against TBEV, JEV, and YFV (see Table 1); it is noteworthy that no human vaccine against WNV has yet been developed [37]. WNV can induce severe neurological symptoms in older or immunosuppressed patients, which renders vaccine development more challenging since the safe and efficient introduction of vaccinal antigens into weakened immune systems requires innovative strategies [95]. Because extensive reviews on WNV vaccines on the market and in development have been published recently [4,96], this review will focus on the vaccination programs against flaviviruses currently in place in Europe and the regional differences they demonstrate. The objective is to highlight expected sources of variation when serologically screening humans or animals for WNV. Indeed, up to now, no diagnostic tool has been able to distinguish between naturally infected and vaccinated people or animals, and thus vaccination history should be carefully considered by clinicians and diagnosticians when interpreting the serological results of flavivirus screening tests. 

In the case of WNV, only veterinary vaccines are currently used in Europe and have proven to be very effective in protecting horses from meningoencephalitis in North America [97]. While four vaccines are currently available in the U.S. (two inactivated, one DNA, and one canarypox recombinant, see Table 1), only one of the inactivated vaccines (EQUIP^®^ WNV, formerly Duvaxyn^®^ WNV, Fort Dodge Animal Health/Pfizer) and the canarypox recombinant vaccine (PROTEQ WEST NILE^®^, Merial/Sanofi Aventis) have been commercialized in Europe (since 2009 and 2011, respectively). Immunization schedules involve administrating two doses of vaccine 3 to 6 weeks apart and annual boosters before the WNV transmission season (occurring in August–October in Europe for mammalian hosts). The emergence and pathogenicity of lineage 2 WNV strains in Europe has put into question the ability of existing WNV vaccines, which were developed from North American lineage 1 isolates, to provide protection against European WNV isolates. Both inactivated and canarypox vaccines have been shown to protect against virulent lineage 2 strains in immunochallenged mice and horses, respectively [98,99]. It should be noted, however, that since WNV outbreaks in horses in Europe have been limited and unpredictable, the two WNV vaccines have been used sparingly in European countries, predominantly in Italy and in Hungary (S. Lecollinet, pers comm.). 

Outside of Europe, WNV vaccination programs have been implemented to protect highly susceptible domestic bird species, e.g., geese (inactivated vaccine available in Israel [100]) and endangered bird species (DNA vaccine in California condors (*Gymnogyps californianus*) [101] or inactivated and canarypox recombinant vaccines commercialized for horses used in scrub-jays (*Aphelocoma insularis*) [102]. DNA vaccines have been also used to experimentally reduce WNV viremia in bird reservoir hosts, such as crows (*Corvus brachyrhynchos and ossifragus*) [103,104] and American robins (*Turdus migratorius*) [105], in the lab. The latter species is considered to play a key role in virus amplification in the U.S. Nonetheless, to our knowledge, birds have never been vaccinated against WNV, nor against other flaviviruses, in Europe. 

In humans, YFV and JEV vaccines are recommended for European travellers visiting areas in which the viruses are endemic, e.g., tropical and subtropical regions of Africa and South America or Southeast Asia, respectively. Vaccination against YFV is still based on the highly effective and relatively safe 17D live-attenuated vaccines initially developed in 1937 by Max Theiler [37,106]; protection lasts for at least 10 years. Inactivated and live-attenuated vaccines are also available for the immunoprophylaxis of Japanese encephalitis (Table 1). Inactivated vaccines were originally derived from infected primary cells (mouse primary cell cultures), but the IXIARO^®^ vaccine, based on the formalin-inactivated attenuated SA14-14-2 JEV strain grown in Vero cells, has demonstrated excellent safety and immunogenicity profiles; it is now the only JEV vaccine licensed in Europe, the U.S., or Australia [107,108]. In the case of TBEV, the epidemiological situation is different. TBEV circulates endemically in Europe and causes more than 10,000 severe cases requiring hospitalisation every year. TBEV vaccination of people with a high risk of exposure should therefore be encouraged. 

**Table 1:** Types of flavivirus vaccines approved for use in target species (humans and horses) in Europe or elsewhere in the world if not available in Europe.

	Virus	Vaccine form	Antigen
Human vaccine	JEV	Inactivated vaccine Ixiaro^®^—licensed in Europe, JE-VAX^®^—commercialization stopped	Whole virus
		Attenuated JEV vaccine (strain SA14-14-2) Available only in China and South Korea	Whole virus
		Chimeric vaccine combining the YFV non structural (NS) proteins and the JEV prM-E Chimeri Vax-JE^®^ Available only in Australia and Thailand	Precursor membrane-Envelope (prM-E)
	TBEV	Inactivated vaccine Ticovac^®^ or Encepur^®^ or FSME-Immune^®^—licensed in Europe TBE Moscow vaccine^®^ and EnceVir^®^—licensed in Russia strain from Brazil); attenuated by passage through embryonated eggs Stamaril^®^ or YF-VAX^®^—licensed in Europe and the rest of the world	Whole virus
YFV	Live-attenuated vaccine (Rockefeller 17D strain or 17DD strain from Brazil); attenuated by passage through embryonated eggs Stamaril_®_ or YF-VAX_®_—licensed in Europe and the rest of the world	Whole virus
Horse vaccine	JEV	Inactivated vaccine Nisseiken^®^ Available only in Japan	Whole virus
	WNV	Inactivated vaccine + adjuvant West Nile-Innovator^®^ (U.S.) or Vetera^®^ WNV (U.S.) or EQUIP^®^ WNV (Europe)	Whole virus
		Chimeric recombinant canarypox virus Recombitek^®^ Equine WNV (U.S.), Proteq West Nile^®^ (Europe)	prM-E
		Chimeric vaccine combining YFV NS proteins and WNV prM-E PreveNile^®^ Available only in the U.S., recalled in 2010	prM-E
		DNA vaccine + adjuvant West Nile-Innovator^®^ DNA Available only in the U.S.	prM-E

TBE can be effectively prevented in the field using purified inactivated whole virus vaccines that are produced in Europe and Russia from cell cultures (such as FSME-IMMUN^®^ or Encepur^®^, manufactured in Austria and Germany, respectively) and that are administered over the course of a comprehensive vaccination program that comprises multiple boosters. Their use varies widely in areas in which the virus is endemic, with high vaccination coverage in Austria (88%) and in the Sverdlovsk region of Russia, where a mass immunization program was initiated in 1996 [37,109]. 

In general, Europe has experienced complex and varied situations with regards to the natural occurrence of flaviviruses and flavivirus vaccination programs, which complicates the use of WNV serological diagnostic tools. 

## 3. Serological Cross-Reactions between West Nile and Related Flaviviruses

Flaviviruses are antigenically related, as originally shown by HITs [110], ELISAs, or VNTs [45]. Although broad serological cross-reactions in general and cross-neutralizations in particular are observed and allowed defining serocomplexes, their extent and duration are strongly dependent on amino acid similarity in a few viral proteins [5,111]. 

### 3.1. Viral Determinants of Serological Reactions

**Figure 3:**
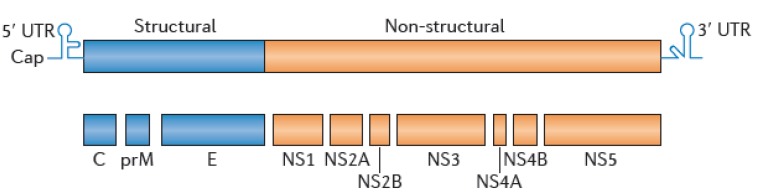
Organisation of the flavivirus genome.

Flaviviruses consist of enveloped and spherical particles surrounding a ssRNA+ genome of about 11 kb (Figure 3). The flavivirus genome comprises a single open reading frame that codes for three structural proteins, the envelope protein (E), the precursor membrane (prM), the capsid (C), and at least seven nonstructural proteins (NS1, 2A, 2B, 3, 4A, 4B and 5).

The immunodominant antigens of the viral particle are the E, the prM, and NS1 proteins, and most serological tools rely on the detection of anti-E and/or anti-NS1 antibodies. The major neutralizing determinants are present in the E protein [112]. Upon folding, the E protein has three structural domains, DI, DII, and DIII, which are separated by short flexible hinges and organized as antiparallel homodimers in mature virions. As a result of this conformation, antibodies can more readily access the outer or lateral surfaces but have a harder time reaching the inner surfaces [113]. The center of DI connects with both DII, which is an elongated finger-like domain that contains a highly conserved hydrophobic fusion loop and is involved in endosomal membrane fusion, and DIII, which is an immunoglobulin-like domain that mediates virus attachment to host cells [114,115].

DI and DIII contain virus-specific epitopes, as evidenced in a study of monoclonal antibodies that found that certain consensus epitopes are common to WNV strains but are distinct in other flaviviruses [116]. The epitopes recognized by broadly cross-reactive antibodies have been mapped to the fusion peptide loop located at the tip of DII, a region that is highly conserved among all flaviviruses [117,118]. DIII is also recognized by strongly neutralizing antibodies, and WNV resistance to antibody-mediated neutralization was correlated with mutations in DIII epitopes [116,119]. However, the titer of antibodies against DIII does not necessarily correlate with the clinical outcome in human patients [120]. Conversely, the fusion loop of DII, which is cryptic in mature virions, does not contribute to flavivirus neutralization [120,121]. Nevertheless it has been shown in rodent models that protection against WNV can be afforded by poorly neutralizing anti-DII antibodies [120,122], presumably because their Fc portions interact with complement factors and with Fc receptors on monocytes and macrophages [122,123].

The NS1 protein also participates in protective immunity against flaviviruses. It is unique in that it is the only glycoprotein that is secreted by flavivirus-infected cells and is also exposed at the surface of infected cells [124,125]. Protective immunity is believed to result when anti-NS1 antibodies bind to NS1 proteins associated with the cell surface, which facilitates the phagocytosis by activated Fc-γ receptors and clearance of infected cells [116,126]. 

### 3.2. WNV Serological Diagnostic Tools

**Figure 4:**
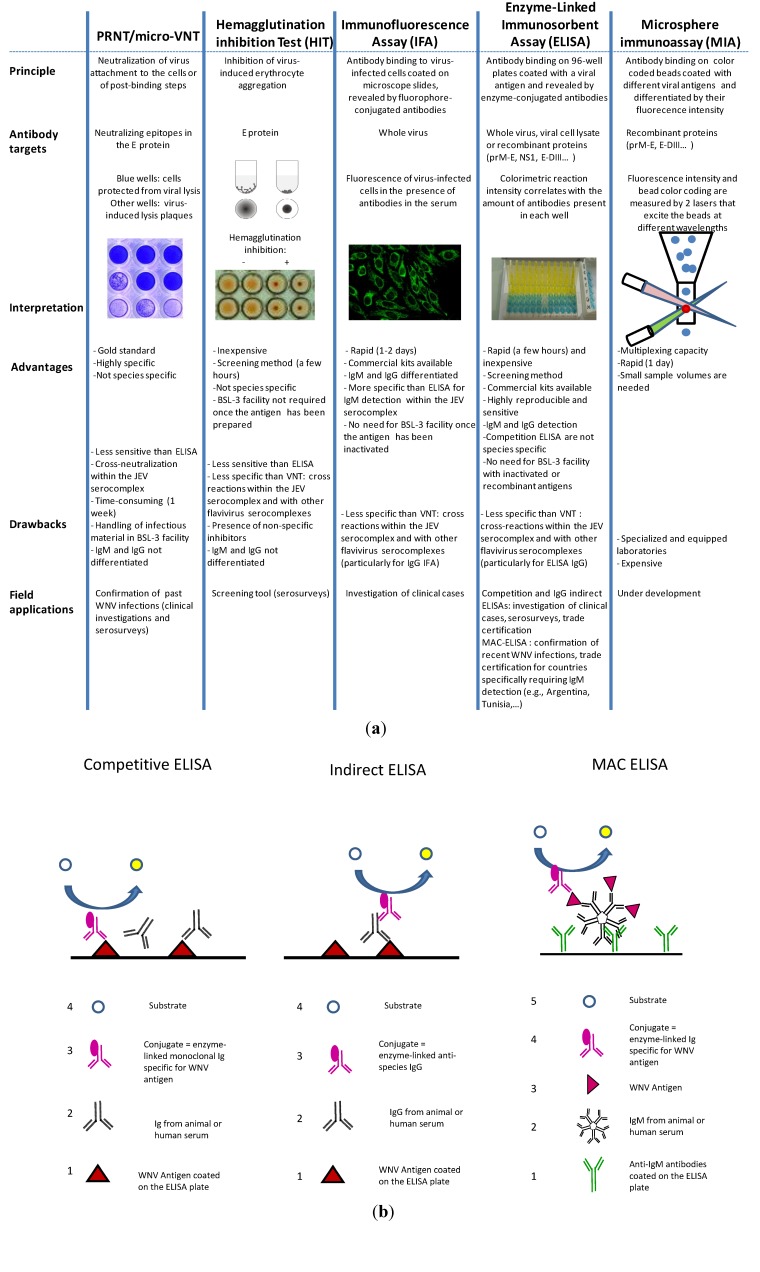
(**a**) Main serological tests used to diagnosis WNV (**b**) Illustrations of the three different ELISA methods: Competitive, indirect IgG, and MAC (the numbers indicate step order).

Many diverse serological tools are available to diagnosis or screen for WNV antibodies [127]. The most commonly used are VNTs (PRNTs or micro-VNTs), IFAs, and ELISAs. The principles underlying these methods as well as their advantages and drawbacks are summarized in Figure 4(a). 

#### 3.2.1. PRNT/Micro-VNT

The gold standard WNV serological test is still the plaque reduction neutralization test, which is performed according to standard protocols with a threshold plaque reduction level of 90% (PRNT90) [128,129]. In this test, the ability of antibodies to reduce lysis plaque number in cell cultures is visually quantified; a sample is considered seropositive if the threshold level (relative to the control) is attained. The micro-virus neutralization test (micro-VNT) is a modification of the PRNT and allows a larger number of samples to be screened using cell microplates. 

Overall, VNTs are time consuming and require that infectious virus be handled in Biosafety Level 3 (BSL-3) facilities or that a chimeric flavivirus such as the YFV/WNV recombinant virus (ChimeriVax^®^ WN) be handled in BSL-2 facilities. They have a higher level of specificity but are less sensitive than ELISAs [130], and their sensitivity may depend on the WNV strain involved [131]. 

#### 3.2.2. Hemagglutination Inhibition Tests (HITs) and Complement Fixation Tests (CFTs)

HITs are still used in some laboratories, whereas CFTs are hardly ever performed because it is difficult to maintain the satisfactory quality controls they require [68]. HITs exploit the ability of the E protein to aggregate avian erythrocytes in the absence of anti-E neutralizing antibodies. Test results are affected by the presence of non-specific inhibitors [132]. HITs are less sensitive than ELISAs and present the same drawbacks, e.g., a high level of antibody cross-reactivity [130,133]. 

#### 3.2.3. Immunofluorescence Assays

IFAs use slides coated with flavivirus-infected cells. Serum samples are deposited on the slides, and the attachment of antibodies is revealed using fluorophore-conjugated immunoglobulins that demonstrate specificity to anti-species IgGs or IgMs. This method is fast and easy to perform and may be used to differentiate between IgM and IgG antibody responses. However, it is not adapted for screening purposes. IFA slides coated with WNV, JEV, YFV, and DENV are commercially available for the diagnosis of human infections [39]. BSL-3 facilities are not required because inactivated virus is bound to the slides. When dealing with antibodies against viruses within the JEV serocomplex, IFAs demonstrate greater specificity in detecting IgMs than do ELISAs; in contrast, IFAs show the same level of specificity as competitive and IgG indirect ELISAs when detecting IgGs [39,134].

#### 3.2.4. ELISA

ELISAs are preferred screening tools because of their rapidity, sensitivity, reproducibility, and affordability. Three different assays are commonly used: The competitive ELISA, the indirect IgG ELISA, and the IgM antibody-capture (MAC) ELISA (Figure 4(b)). Ready-to-use diagnostic kits for veterinary and human purposes are commercially available. 

The MAC-ELISA is suitable for detecting acute infections (8–60 days post-infection) in humans and horses; however, its usefulness in determining acute infections is questionable in humans, in whom WNV IgMs have been reported occasionally to persist for over a year [4]. This test is a highly valuable tool because IgM antibodies are hard to detect using VNTs or HITs [133,135] and exhibit less cross-reactivity than IgG antibodies [136,137]. 

Indirect IgG ELISAs require the use of adapted anti-species conjugated antibodies [4,138]. Because of the wide variety of WNV hosts, veterinary diagnostic laboratories are gradually replacing indirect IgG tests with competitive (or blocking) ELISAs, which allow the testing of samples from virtually all animal species [139]. This versatility relies on the ability of WNV antibodies present in the sample to compete for antigenic binding sites with WNV-specific monoclonal antibodies. Competitive ELISAs are the most sensitive of all the developed serological technologies, but they are most appropriate for screening purposes because of their lower level of specificity. 

The sensitivity and specificity of ELISAs are strongly influenced by the nature of the bound antigen. Attempts have been made to improve ELISA specificity using a recombinant version of the DIII of the E protein [140,141] or by substituting NS1 [142] for the whole virus or prM-E antigens. E-DIII ELISAs are able to differentiate between infections caused by flaviviruses belonging to JEV or TBEV serocomplexes [140] as well as between those caused by WNV and other flaviviruses belonging to the JEV serocomplex, such as JEV, MVEV, and SLEV [141]. NS1 ELISAs can differentiate between infections caused by WNV and those caused by JEV or SLEV in animals and do not react with sera originating from animals vaccinated with most DNA or recombinant WNV vaccines (*Differentiating Infected from Vaccinated Animals*—DIVA*—*strategies) [96,142,143].

#### 3.2.5. Microsphere Immunoassays

Microsphere immunoassays are potentially useful tools because of their multiplexing capacity; they couple different flavivirus antigens to fluorescent and differentiable beads [144]. Nevertheless, cross-reactivity within the JEV serocomplex among antibodies targeting the E antigen occurs as frequently in microsphere immunoassays as in ELISAs, and specificity thus needs to be improved. 

In conclusion, the specificity of many serological tests is still an issue of major concern. High-throughput screening tests that perform at high level of specificity are crucial, and recent improvements have been made in this field [96]. The improved selection of relevant epitopes from a wider range of monoclonal antibodies that target different flaviviruses will surely help determine the precise antigenic sites that differ among these flaviviruses and will thus ultimately lead to the development of more specific diagnostic tests [145]. 

### 3.3. Misinterpretation in the Diagnosis and Surveillance

Serological cross-reactivity may lead to the misinterpretation of diagnostic test results obtained from suspected clinical cases and samples collected during WNV epidemiological serosurveys. Awareness of possible cross-reactions is essential given that the aim of such investigations is to correctly identify the viruses responsible for the observed serological reactions. The nature and prevalence of cross-reactions differ depending on the target species and geographical location. Overall, the risk of cross-reactions exists wherever multiple flaviviruses co-circulate, including in Europe.

#### 3.3.1. Difficulties for Accurate Diagnosis

The serological diagnosis of WNV infection is complicated by the fact that the patients or animals may have previously been exposed to or vaccinated against cross-reacting viruses. In this context, it is essential to use tests that are both sensitive enough to detect WNV infection and specific enough to distinguish an acute WNV infection from one caused by a closely related virus or remnants of prior WNV exposure. Here, we report the results of field and experimental studies that have compared diverse serological methods with the objective of optimizing the specificity of the serological diagnosis of WNV infection. 

##### Use of ELISAs

ELISAs are the most commonly used diagnostic assays because of their simplicity and relatively low cost; they also require few specialized apparatus and facilities. When WNV first emerged on the North American continent in 1999, samples of serum and cerebrospinal fluid from the first eight patients hospitalized with viral encephalitis and meningitis tested positive for anti-SLEV antibodies based on IgM-capture and indirect IgG ELISAs; SLEV is a common flavivirus that is an enzootic of North America [146]. However, doubt remained as to whether SLEV was the etiologic agent because the clinical manifestations were not those typically associated with SLEV infection, polymerase chain reactions using SLEV-specific primers yielded negative results, and serological testing using a SLEV-specific PRNT revealed only low levels of neutralizing antibodies [146,147]. WNV was later identified as the cross-reactive etiologic agent [148]. 

Similar misinterpretations of serological results may occur in Europe because those being tested may have been exposed to co-circulating or newly emerging flaviviruses or have encountered flaviviruses while traveling. For example, although successive experimental infections in horses have suggested that WNV-specific competitive ELISAs using anti-NS1 MAb 3.1112G as a conjugate are highly specific for WNV [149], high rates of false-positives were obtained for human sera from patients with confirmed DENV infections [150]. This serological method therefore cannot be used to accurately diagnose WNV infections in humans in areas where other flaviviruses, such as DENV, are endemic. Similarly, during the 2010 WNV outbreak in Greece, commercially-available ELISA kits were used to test for IgMs and IgGs against WNV, DENV, and TBEV in sera and cerebrospinal fluid sampled from patients suffering from encephalitis or meningitis. A high degree of cross-reactivity was observed between WNV and DENV IgMs, although the WNV-specific antibodies clearly demonstrated a stronger serological response [151]. In this study, none of the patients were vaccinated against JEV, YFV, or TBEV. Knowing patient vaccine history is important when interpreting serological results because vaccination may interfere with the accurate diagnosis of acute WNV infection in humans and horses. For instance, Shirafuji *et al.* [152] showed, using WNV and JEV MAC-ELISAs, that horses experimentally infected with WNV tested negative for WNV IgM antibodies if they had previously been vaccinated against JEV, whereas WNV IgM antibodies were detected in the non-vaccinated group. In addition to vaccination records, the travel history of each patient needs to be considered because WNV serodiagnosis should take into account the possibility of prior exposure to naturally circulating flaviviruses [39]. 

Other methods, such as IFAs that detect IgMs or ELISAs that use virus-specific epitopes such as rEDIII, may prove to have greater specificity than above-mentioned ELISAs. However, studies consistently discourage basing a flavivirus serodiagnosis on single assay and only searching for a single virus [39]. Furthermore, if multiple flavivirus infections or vaccination against other flaviviruses are likely, then repetitive sampling is of great importance, as the etiological virus could be misdiagnosed if only a single sample is tested [149]. Even though it is difficult to do in the field, physicians and veterinarians are strongly encouraged to collect both acute and convalescent sera, spaced about 2–3 weeks apart, in order to pick up on seroconversions and identify false negatives that result from delayed IgG and IgM responses in individuals with acute WNV infections. Overall, this level of complexity in flavivirus diagnosis likely leads to an underestimation of the incidence of acute WNV infections worldwide. 

##### Use of Virus Neutralization Tests 

As mentioned earlier, VNTs have a high degree of specificity for target flaviviruses, but cross-neutralization by antibodies against viruses within the same serocomplex is still observed. As a result, a thorough comparison of the end point titers obtained using other flaviviruses known to be endemic in the area should be performed [45,153]. Virus identification thus relies on finding the virus associated with the highest neutralization titers, provided that said titers are at least fourfold higher than those associated with the second-ranked virus; however, difficulties may nonetheless arise if two consecutive flavivirus infections take place [154]. 

As a consequence, diagnostically differentiating among viruses may be hard in areas in which flaviviruses are hyperendemic [155]. In particular, it has been experimentally shown that horses previously vaccinated against JEV had higher neutralizing antibody titers against both JEV and WNV after a WNV challenge and that their levels of JEV-neutralizing titers were higher than or equal to their levels of WNV-neutralizing titers [152].

#### 3.3.2. Challenges for WNV Surveillance Programs

The use of tests with high levels of specificity is even more important in WNV serological surveillance because the expected prevalence in the target population is lower and therefore the risk of obtaining false positives is higher. Another technical constraint is the necessity to rapidly and simultaneously screen large numbers of samples; this need makes ELISAs the method of choice since they are inexpensive and safe to perform under minimal biocontainment conditions. However, confirmation of sample seropositivity using VNTs is required to specifically identify the virus(es) responsible for the observed serological reactions. Because PRNT90 lacks sensitivity in weakly exposed populations, some authors have suggested that PRNT50 should be used in serological surveys; however, this latter test is not reliable enough to obtain robust conclusions [131,156,157]. 

In humans, serosurveys most often rely on sera collected from healthy blood donors whose vaccination and travel histories are unknown even though such factors may strongly affect the results of the serological tests used to detect WNV-specific antibodies. For example, in North Tyrol, initial ELISA results suggested that WNV seroprevalence was 46.2%; however, comparative neutralization tests revealed the existence of cross-reactions with anti-TBEV antibodies or other vaccine-associated flaviviral antibodies, which generated false positives. Accounting for these false positives, WNV seroprevalence was found to be 0.5% instead [158]. In particular, it has been shown that people vaccinated against YFV are more likely to neutralize WNV than those not vaccinated against YFV [38].

Estimating the likelihood of encountering cross-reactions is particularly important in studies that use animal sentinels such as horses or captive birds to rapidly detect WNV circulation [26,156,159,160]. Depending on the geographical location, serological diagnostic algorithms may help differentiate WNV from other, co-occurring flaviviruses [154]. 

The results of serosurveys of free-ranging birds are particularly complex to interpret because some species are long-distance migrants that may encounter WNV and other flaviviruses at a large variety of exposure sites [87,161]. However, sedentary bird species in Europe may also be exposed to a wide diversity of flaviviruses [16,67,78]. Parallel titration for antibodies against several flaviviruses using VNTs is therefore essential in identifying exposure to potentially emerging flaviviruses and is even more crucial in assessing the continued transmission of introduced flaviviruses in Europe [68,76,85,132,162,163,164]. Similar constraints exist in serosurveys of wild ungulates because the diversity of the flaviviruses that may circulate in these species is poorly known [165]. 

Determining the age of seropositive hosts is of the utmost importance in WNV serosurveys, even if age can only be estimated, as is the case for wild animals. Younger animals should be excluded from immunological analyses because the presence of maternal antibodies in their systems could lead to a misinterpretation of the results. Maternal antibodies against WNV have been shown to remain detectable in the serum of horses for up to 120 days [166] and of birds for up to 9 days in passerines [167] and 42 days in domestic chicken [168]. If young animals that are old enough to have cleared maternal antibodies are seropositive, it suggests that recent transmission has occurred within a group or population, and the results may thus help identify the time period during which the virus was circulating [76,132]. Furthermore, enzootic or epizootic circulation patterns can also be inferred by analyzing seroprevalence across different age classes; enzootic transmission should be associated with increased seroprevalence rates in older individuals [19,169]. 

As previously mentioned, the level of serological cross-reactivity frequently observed among flaviviruses can lead to diagnostic misinterpretation, but it could also have a beneficial effect by ensuring partial or complete cross-protection against serial flavivirus infections. On the other hand, it could also facilitate subsequent flavivirus infections via a mechanism called antibody-dependent enhancement (ADE), which has mainly been described for the dengue flavivirus but is possibly applicable to other flaviviruses.

## 4. Antibody-Dependent Cross-Protection or Enhancement among Flaviviruses

Few flavivirus vaccines are currently available for human use, and two in particular are lacking: those against the widespread DEN and WN viruses. It is therefore of utmost interest to evaluate the capacity of flavivirus vaccines to protect against heterologous flaviviruses. Cross-protection can most easily be assessed using vaccination trials that employ appropriate experimental conditions: experiments in which animal models have been chosen, appropriately controlled, and immunologically challenged after initial vaccination or infection with heterologous flaviviruses. Furthermore, using VNTs, the neutralizing capacities of sera obtained from vaccinated target species can be analysed and may also provide the scientific and medical community with valuable information. 

### 4.1. Evaluation of Cross-Protection in Animal Models

Several animal models have been used to assess the level of heterologous protection against flaviviruses; these models include two epidemiologically important bird species, house finches (*Haemorhous mexicanus*) and red-winged black birds (*Agelaius phoeniceus*), as well as four mammal species, Syrian golden hamsters (*Mesocricetus auratus*), Swiss mice (*Mus musculus*), bonnet macaques (*Macaca radiata*) and pigs (*Sus domesticus*). The major results of studies addressing this topic are summarized in Table 2. 

These animal models illustrate that prior immunization with heterologous flaviviruses and a challenge with a virulent strain generally induce immune booster effects, marked by an increase in neutralizing antibodies, a reduction or absence of the viremic phase after challenge, and subsequent complete or partial clinical protection (evaluated only when the challenge flavivirus was pathogenic in the animal species). This protective effect was readily observed for flaviviruses belonging to the same serocomplex.

**Table 2:** Assessment of the heterologous protection afforded by prior flavivirus infection or vaccination in animal models.

	Species (sample size)	Infection or Vaccination	Challenge	Heterologous protection	Publication
Birds	House finches (8)	SLEV Kern217 virulent strain	WNV NY99 virulent strain	Complete clinical protection (0/8 death *vs.* 3/4 deaths in control group), but insufficient virological protection (viremia, mean of 4.6 (2.7–6.4) log_10_ PFU/mL considered sufficient to infect susceptible mosquitoes)	[170]
	House finches (8)	WNV NY99 virulent strain	SLEV Kern217 virulent strain	Sterilizing immunity: absence of viremia (0/8 *vs.* 4/4 in control group)	[170]
	Red-winged blackbirds (8)	WNV NY99 virulent strain	JEV virulent Indian strain (genotype III)	Nearly complete virological protection (1/16 viremic birds *vs.* 16/16 controls)	[171]
	Red-winged blackbirds (8)		JEV virulent Vietnamese strain (genotype I)		
Mammals	Pigs (2)	MVEV virulent OR2 strain	JEV virulent Nakayama strain	Sterilizing immunity: absence of viremia (0/2 *vs.* 1/1 in control group)	[155]
	Pigs (2)	WNV mildly virulent KUN HU6774 strain	JEV virulent Nakayama strain	Sterilizing immunity: absence of viremia (0/2 *vs.* 1/1 in control group)	
	Bonnet macaques (3)	Formalin-inactivated JEV strain (733913)	WNV virulent 68856 strain	Complete clinical protection (0/3 death *vs.* 2/3 deaths in control group)	[172]
	Bonnet macaques (5)	Formalin-inactivated WNV strain (68856)	JEV virulent 733913 strain	Partial clinical protection (1/5 death *vs.* 1/1 death in control group)	
	Hamsters (30)	JEV SA14-2-8 vaccine strain	WNV virulent NY99 strain	Complete clinical protection (0/30 death *vs.* 14/30 deaths in control group). Viremia lowered by about 3 log	[173]
	Hamsters (32)	SLEV virulent Be Ar 23379 strain	WNV virulent NY99 strain	Complete clinical protection (0/32 death *vs.* 14/30 deaths in control group). Viremia lowered by about 4 log	
	Hamsters (30)	YF 17D vaccine strain	WNV virulent NY99 strain	Partial clinical protection (4/30 deaths *vs.* 14/30 deaths in control group). Viremia slightly lowered	
	Hamsters (50)	DENV-2 New Guinea C strain	WNV virulent strain	Partial clinical protection (8/50 deaths *vs.* 50/50 deaths in control group).	[174]
	Swiss mice (29)	DENV-2 New Guinea C strain	JEV virulent Peking strain	Complete clinical protection (0/29 deaths *vs.* 38/60 deaths in control group). Sterilizing immunity: absence of viremia.	[175]
	Swiss mice (45)	DENV-2 New Guinea C strain	SLEV virulent Pinellus P 15 strain	Partial clinical protection (17/45 deaths *vs.* 80/90 deaths in control group).	

### 4.2. Evaluation of Cross-Protection in the Field

Credible data on heterologous protection in the field have mainly been obtained by studying the *in vitro* neutralization capacity of human sera sampled from vaccinated individuals. Mansfield *et al.* [38] used PRNTs to investigate flavivirus cross-reactivity in sera from a human cohort with a history of vaccinations against TBEV, JEV, and YFV. The PRNTs indicated that 64% (16/25), 89% (25/28), and 35% (9/26) of the samples tested contained TBEV, JEV, and YFV neutralizing antibodies, respectively. Neutralization of LIV, DENV-2, and WNV occurred in 88% (22/25), 38% (10/26), and 53% (15/28) of cases, respectively; in contrast, no significant neutralization of MVEV was observed. Vaccination history modified WNV cross-neutralization efficacy: individuals that had received the three vaccinations had an increased chance of being WNV-neutralizing-antibody positive than individuals vaccinated against TBEV and JEV alone. 

It was clear that, in humans, vaccination against TBEV and JEV could elicit an antibody response capable of limited WNV neutralization. Although this study did not control for variables such as the age of the subjects or the time between YFV vaccination and blood sampling, the results suggested that the additional vaccination against YFV enhanced the ability of sera to neutralize WNV [38]. This finding differs from that of a previous study that concluded that JEV vaccination was more effective than YFV vaccination at eliciting WNV cross-protection through cross-reactive antibody responses [176]. Current vaccines against YFV are live-attenuated rather than inactivated and are thus likely to be more immunogenic, stimulating a broader spectrum of responses and inducing a strong humoral and cell-mediated immune response. Interestingly, a recently published preclinical assessment of JE-ADVAX®, a new candidate JEV vaccine that consists of an inactivated JEV strain and a delta inulin adjuvant, suggested that the vaccine was capable of inducing robust cross-protective immunity against a virulent WNV strain used to challenge a murine model [177]. This study and others testing the antigenicity of inactivated and adjuvanted or non-adjuvanted JEV vaccines (JE-VAX^®^) compared to that of live-attenuated JEV vaccines (the SA-14-14-2 strain used in China) stress the importance of adjuvant formulation in the induction of high-quality heterologous immunity by inactivated flavivirus vaccines [123,178]. 

Most individuals vaccinated against TBEV demonstrated a LIV neutralizing response. There is currently no human vaccine available for tick-borne flaviviruses, apart from TBEV, but the results presented by Mansfield *et al*. [38] suggest that vaccination against TBEV may offer partial protection against LIV infection in humans. Moreover, a TBEV vaccine based upon the European prototype Neudoerfl strain was recently shown to induce cross-reactive antibodies in humans, both against other TBEV subtypes as well as against more distantly related flaviviruses, such as OHFV [179].

Cross-reactivity among antibodies against flaviviruses could significantly contribute to the development of effective broad-spectrum human vaccines against WNV and other existing or emerging flaviviruses. ChimeriVax technology has utilized this cross-reactivity to its advantage in the development of vaccine candidates [178,180]. Ultimately, chimeric vaccines that assemble components from different individual flaviviruses would be of great help in countries dealing with different flaviviruses.

The results of all of the studies presented in Section 3.1 and Section 3.2 concur in finding that at least partial immunity to heterologous flaviviruses is afforded by available or candidate vaccines. Nevertheless, since the extent and duration of cross-neutralization and cross-protection are strongly dependent on the degree of amino acid similarity among flaviviruses, it is likely that vaccination against more distantly related flaviviruses will not be as efficient in inducing protection [37]. The following question remains to be addressed: In the case of non-optimal cross-reactions, could vaccination or infection result in ADE-mediated disease exacerbation, as frequently described for DENV? 

### 4.3. ADE Risk

In some instances, the presence of cross-reacting antibodies acquired during a primary infection could increase the infectivity and exacerbate the disease outcome of a second, antigenically-related virus. This phenomenon, called ADE, has been observed *in vitro*, and is still the subject of debate for a few viruses, including *Flavivirus* species such as DENV. The most severe form of the disease, dengue hemorrhagic fever or shock syndrome (DHF/DSS), mainly occurs after two or more consecutive infections with different DENV serotypes (DENV strains are classified into four serotypes, 1–4) [181,182,183] and may be mediated by the interaction of IgG-virus immunocomplexes with Fc-γ receptor-bearing cells, such as monocytes/macrophages, dendritic cells, and endothelial cells, which subsequently become infected [182]. Whether this phenomenon also occurs as a result of consecutive infections with other flaviviruses is still a matter of speculation. On the one hand, experimental studies have shown that the ADE of WNV infections can be induced *in vitro* using rabbit hyperimmune antisera developed against a wide range of flaviviruses, including members of both the JEV and TBEV serocomplexes, whereas the ADE of TBEV infections is only elicited *in vitro* by members of its own serocomplex [184]. On the other hand, evidence of the ADE of flavivirus infections in nature is still lacking. However, obtaining evidence of ADE is not straightforward given that the distribution of many flaviviruses is either geographically [36] and/or ecologically [16] restricted. Nevertheless, as flaviviruses emerge in areas outside of their traditional geographical ranges, the co-circulation of different flaviviruses within the same area is becoming more common. For instance, co-circulation has already been observed in areas of central and eastern Europe, where the recently emerged WNV strain co-occurs with endemic TBEV. A recent report describing an unusually severe TBEV case in a patient in Hungary previously exposed to WNV suggested that the case’s severity could result from ADE [185]. 

As previously mentioned, human vaccines against flaviviruses (JEV, YFV, TBEV) could ensure partial cross-protection against heterologous flaviviruses within the same serocomplex. At the same time, there is limited experimental evidence of ADE subsequent to vaccination and virus challenge. In mice, antibodies elicited by inactivated-JEV vaccines could enhance pathogenesis via ADE when mice were challenged with a heterotypic flavivirus such as MVEV [178]. Consequently, the risk that vaccination will facilitate a subsequent flavivirus infection, although likely to be low, deserves further consideration, and appropriate studies should be carried out to address ADE occurrence *in vitro* and *in vivo* [123].

## 5. Conclusions

European countries are currently facing clinical outbreaks or silent circulation of a wide diversity of flaviviruses, and it is expected that circumstances will become even more complex in the coming years as human travel continues to intensify, the trade of animals and goods facilitate virus emergence through the importation of infected vectors and/or reservoir hosts [88,186], and heretofore unknown flaviviruses that already established in Europe are discovered. WNV diagnosis and surveillance protocols and schemes clearly need to be improved in Europe and across the globe. The specificity of serological laboratory tests should be enhanced and adapted to specific epidemiological patterns. Moreover, serological surveys should be accompanied by complementary analyses, such as mosquito or animal sentinel surveys aimed at collecting infected samples and identifying the molecular diversity of circulating flaviviruses. Recent developments in surveillance systems have been driven by improved cost/efficiency ratios and consist of implementing risk-based surveillance systems [187,188] in limited geographic areas where flaviviruses are likely to occur. 
